# Thermal Decomposition and Stability of Hybrid Graphene–Clay/Polyimide Nanocomposites

**DOI:** 10.3390/polym15020299

**Published:** 2023-01-06

**Authors:** Caroline Akinyi, Jude O. Iroh

**Affiliations:** Department of Materials Science and Engineering, University of Cincinnati, 2600 Clifton Ave., Cincinnati, OH 45221, USA

**Keywords:** graphene, clay, polyimide, nanocomposites

## Abstract

Polyimide matrix nanocomposites have gained more attention in recent years due to their high thermal stability, good interfacial bonding, light weight, and good wear resistance and corrosion, factors that make them find great applications in the field of aerospace and advanced equipment. Many advancements have been made in improving the thermal, mechanical, and wear properties of polyimide nanocomposites. The use of nanofillers such as carbon nanotubes, graphene, graphene oxide, clay, and alumina has been studied. Some challenges with nanofillers are dispersion in the polymer matrix and interfacial adhesion; this has led to surface modification of the fillers. In this study, the interaction between clay and graphene to enhance the thermal and thermal-oxidative stability of a nanocomposite was studied. A polyimide/graphene nanocomposite containing ~12.48 vol.% graphene was used as the base nanocomposite, into which varying amounts of clay were added (0.45–9 vol.% clay). Thermogravimetric studies of the nitrogen and air atmospheres showed an improvement in thermal decomposition temperature by up to 50 °C. The presence of both fillers leads to increased restriction in the mobility of polymer chains, and thus assists in char formation. It was observed that the presence of clay led to higher decomposition temperatures of the char formed in air atmosphere (up to 80 °C higher). This led to the conclusion that clay interacts with graphene in a synergistic manner, hence improving the overall stability of the polyimide/graphene/clay nanocomposites.

## 1. Introduction

Polyimide is an engineering polymer that finds wide applications in the fabrication of aircraft structures and microelectronic devices. It has excellent chemical, thermal, and mechanical properties [1]. In recent years, a lot of advancements have been made in developing polyimide nanocomposites, because these composites exhibit high strength, better thermal stability, light weight, corrosion resistance, good interfacial bonding, solvent resistance, and antiwear [2]. In developing new polyimide nanocomposites, the focus is on the improvement in thermo-oxidative stability, resistance to microcracking, and the development of a toughened system [3]. These enhancements enable the polymer to meet extreme requirements in some special situations. To enhance the performance of polyimide for engineering applications, various types of nanofillers such as carbon nanotubes, graphene, inorganic oxides, carbon fibers, clay, molybdenum disulfide, ionic liquids, aramid fibers, and boron nitride, among others, have been incorporated into the matrix.

The incorporation of small loadings of nanoscale filler materials in polymers leads to substantial property enhancements compared with the conventional micro- and macroscale composites [4,5]. The small loadings also lead to low component weight and simplified processing [3]. The enhancement in mechanical properties, heat resistance, flammability, and gas permeability with the incorporation of these nanofillers opens a new frontier for the application of polymer nanocomposites.

Among the various nanofillers, graphene has been widely used due to its exceptional thermal, mechanical, and electrical properties [6,7,8,9]. Its availability in bulk quantities as colloidal dispersion and powders coupled by its relatively easy production via reduction in graphene oxide has made this material particularly attractive. Graphene is a 2D, single-layer nanosheet of sp^2^ hybridized carbon atoms [10,11]. Graphene has been used to significantly improve the mechanical and thermal properties of graphene/epoxy composites [12,13,14]. Graphene is advantageous over carbon nanotubes because of its large surface area and ease of modification by different molecules [15].

Liu et al. investigated the gas barrier and thermal properties of polyimide/graphene and polyimide/clay nanocomposites. They reported decreased water vapor transmission rates and improvement in the temperature of decomposition at 5% weight loss, concluding that addition of these nanofillers improved the thermal stability of the polyimide [16,17]. Wang et al. observed an improvement in the mechanical, thermal, and hydrophobic properties of amino-functionalized graphene oxide/polyimide composite films.

Polymer/layered silicate nanocomposites have attracted attention because of the improvement in material properties when compared with the virgin polymer. These include increased strength and heat resistance [4,18], high moduli [6,19,20,21,22], decreased flammability [5,23,24] and gas permeability [25,26,27], and increased biodegradability of biodegradable polymers [18]. Polymer clay nanocomposites (PCN) have been studied since 1985, with the nylon 6–clay hybrid being the first PCN. Silicate layers of clay were homogenously dispersed into a polymer matrix, which led to superior mechanical, thermal, and barrier properties compared with the pristine and conventional composites. The first commercial application of PCN was in 1989, when cars equipped with nylon 6–clay hybrid were launched by Toyota [19]. Montmorillonite is the most common and ubiquitous clay mineral, and it undergoes swelling and intercalation in the presence of water and organic cations. Agag et al. studied the thermal and mechanical properties of polyimide–clay nanocomposites. They reported a 110% increase in the tensile modulus upon the addition of 2 wt.% organomodified montmorillonite clay to polyimide matrix. They also reported higher decomposition temperatures for the polyimide/clay nanocomposites compared with the original polyimides [28].

Various property enhancements due to the addition of graphene and clay nanoplatelets have been studied and reported. The thermal stability studies have mostly focused on the temperature at onset of decomposition as a determinant factor for the improvement or lack thereof in thermal stability. However, few/no studies exist that delve further into the mechanisms of decomposition of these polyimide/graphene or polyimide/clay nanocomposites. Factors such as the effect of the nanofillers on the rate of decomposition of the polymer and the nature and mechanism of char decomposition have not been thoroughly investigated. These are important factors in elucidating the mechanism of flame retardancy.

In previous research work by this group, polyimide/graphene nanocomposites containing 10–50 wt.% graphene were fabricated, and various properties were reported [29,30,31]. The optimum concentration of graphene in polyimide was determined from these studies, and varying concentrations of clay were added to the polyimide/graphene to study the interactions between graphene and clay and the resulting properties of the hybrid system [32,33]. In this current research work, the focus of the study was in elucidating the decomposition mechanism of the hybrid system and uncovering any synergistic interactions between clay and graphene.

## 2. Materials and Methods

Nanographene sheets of 98.48% purity were purchased from Angstron Materials, Dayton, OH, USA. The graphene sheets were 70–100 nm thick, with a lateral dimension of 2–7 µm. Montmorillonite organomodified Cloisite 30B clay of 100–200 nm thickness and 2–13 µm length was purchased from Southern Clay Products, Inc., Gonzales, TX, USA. Ammonium ion (III) was used in the Cloisite 30B, as the exchange cation is methyl, octadecyl, and bis-2-hydroxyethyl ammonium ion. 4,4-oxydianiline (ODA-98% purity), pyromellitic dianhydride (PMDA-99% purity) and N-methyl-pyrrolidone (NMP-99% purity) were purchased from Sigma Aldrich, St. Louis, MO, USA.

### 2.1. Synthesis of Polyimide–Graphene–Clay Composites

The fabrication of the composites occurred in three stages. The first step involved the synthesis of the poly(amic acid)–graphene solution. The second step was the synthesis of the poly(amic acid)–clay solution. In each case, 5.2 g of ODA was added to 100 mL of NMP contained in a round bottomed flask, followed by mechanical stirring until the solid completely dissolved in the solvent. For the poly(amic acid)–graphene solution, a graphene quantity corresponding to ~12.48 vol.% of total solids was added to the ODA solution, and stirring continued for 8 h. PMDA was then added to the mixture, followed by stirring for 12 h, the temperature of the reactants being maintained at 10 °C. For the poly(amic acid)–clay solution, varying concentrations (0.45–9 vol.%) of clay were added to each solution, and the rest of the process was similar to that of the poly(amic acid)–graphene synthesis procedure. These solutions were then mixed in varying concentrations of clay (0–9 vol.%), while maintaining the amount of graphene at ~12.78 vol.%. The poly(amic acid)–graphene–clay mixtures were cast onto a glass substrate, followed by curing in a vacuum at 120 °C for 2 h and 200 °C for 1 h to obtain PI/graphene/clay composite films (PI–NGS–Clay).

### 2.2. Thermogravimetric Analysis, TGA

Thermogravimetric analysis was used to investigate the mechanism of decomposition and thermal stability. The films were subjected to heating rates of 30 °C/min from 25 °C to 1000 °C in air and nitrogen in a TGA Q50 Thermal Analyzer purchased from TA Instruments, New Castle, DE, USA.

### 2.3. Differential Scanning Calorimetry, DSC

The films were subjected to heating rates of 10 °C/min from 25 °C to 725 °C in nitrogen atmospheres in a DSC Q20 thermal analyzer purchased from TA instruments, New Castle, DE, USA

### 2.4. Scanning Electron Microscopy, SEM

Environmental Scanning Electron Microscopy (ESEM), model FEI XL30 FEG (FEI Company, Hillsboro, OR, USA), was used to study the morphology of the composite films. The samples were coated with silver to improve their conductivity.

## 3. Results

### 3.1. Scanning Electron Microscopy

**Figure 1:**
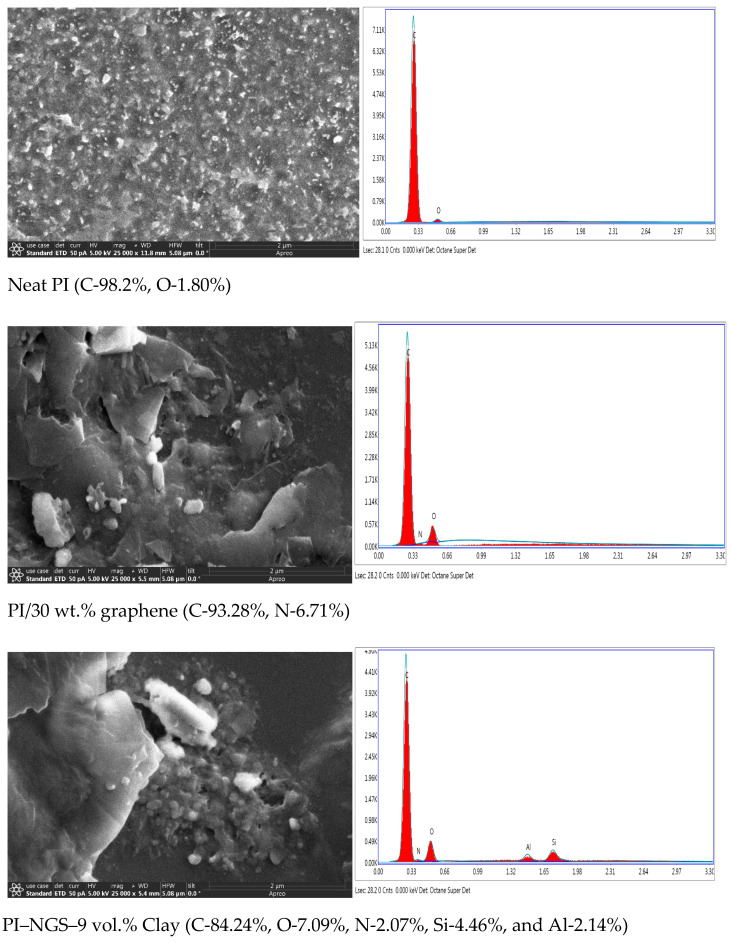
SEM images with EDAX for (top) neat PI, (middle) PI-NGS-0 vol.% clay, and (bottom) PI-NGS-9 vol.% clay, at a magnification of ×25,000.

Scanning electron microscopy with EDAX was used to obtain images of the decomposed char in nitrogen and its elemental composition. This is shown in Figure 1 below. The images were obtained at a magnification of ×25,000. The surface of neat PI char has fine char grains, while the nanocomposites exhibit bulkier structures with uneven and highly coarse surfaces. This is because of the presence of the un-degraded or partially degraded graphene and clay fillers. This is also evidenced in the elemental composition of the char. The clay sample has small quantities of silicon and aluminum, which are elemental constituents of the pristine clay. The neat PI is almost fully degraded, as the char consists of 98% carbon. Lower percentages of carbon are observed in the nanocomposite samples, with the clay sample having 84% carbon in the char. The absence of nitrogen in the elemental composition of neat PI char indicates complete degradation of the imide ring. However, the presence of nitrogen in the PI/graphene and PI/graphene/clay samples indicates some level of protection of the imide ring by the nanofillers from complete degradation.

### 3.2. Thermogravimetric Analysis

#### 3.2.1. Degradation in Nitrogen

**Figure 2:**
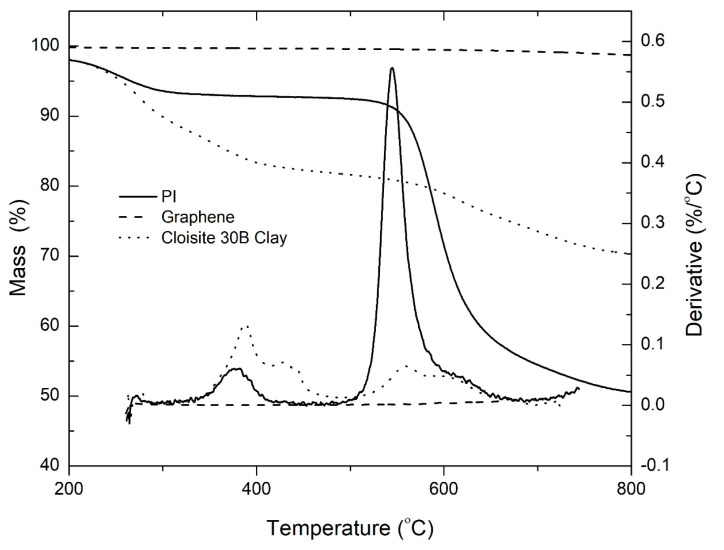
TGA thermograms for neat polyimide, graphene, and Cloisite 30B clay in nitrogen atmosphere at a heating rate of 30 °C/min.

The thermogravimetric degradation profiles of neat PI, graphene and Cloisite 30B clay are shown in Figure 2. The initial decomposition of polyimide occurs between 200 °C and 400 °C. This is attributed to the release of chemically bound water and evaporation of solvent [34]. The polyimide matrix undergoes 40% mass loss in the temperature range of 500–630 °C. Graphene undergoes negligible degradation, as it has been shown to be stable to temperatures above 800 °C in inert atmosphere [35]. Multiple degradation stages are observed on the montmorillonite clay. As previously reported by Tiwari et al. [36], the first degradation stage corresponds to the degradation of the organic modifiers present in OMMT. This starts at 170 °C and peaks at 276 °C and 360 °C. The onset of the third degradation stage occurs at approximately 500 °C and splits into two peaks: one at 540 °C and the second at 620 °C. This can be attributed to the dehydroxylation of the aluminosilicate lattice that leads to the release of structurally bound water [37,38,39]. Carbonaceous char is formed at temperatures above 700 °C. The total mass loss is approximately 30%.

**Figure 3:**
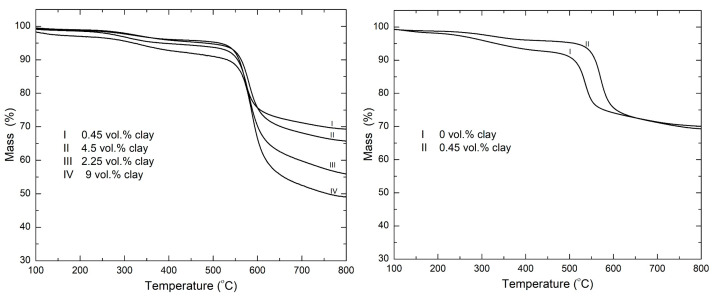
TGA mass loss profiles of PI-NGS-Clay nanocomposites in nitrogen atmosphere at a heating rate of 30 °C/min.

Figure 3 shows TGA mass loss profiles of PI–NGS–Clay nanocomposites with varying amounts of clay; 0 vol.% clay corresponds to PI–NGS containing ~12.78 vol.% graphene. Akinyi et al. previously reported on the decomposition of PI-NGS nanocomposites of varying compositions of graphene [31]. The choice of 12.78 vol.% graphene is based on the fact that the char retention is high, while maintaining a good dispersibility of the filler in the polymer matrix. At higher graphene loadings, there is poor dispersibility, hence affecting the properties of the polymer.

In the Figure 3, it is observed that the onset degradation temperature shifts towards higher temperatures. This increase in the onset decomposition temperature in polymer/clay nanocomposites was also reported by Blumstein [40], who observed an increase of 40 to 50 °C in the decomposition temperature upon the addition of clay to poly(methyl methacrylate). Improved thermal stability has also been reported by Burnside et al. [41] (polydimethylsiloxane–clay nanocomposite) and Lee et al. [42] (polyimide–clay nanocomposite). Delay in the onset temperature of degradation is particularly important for various applications requiring continuous high-temperature operation. In flammability studies, this is a desirable feature as the delay in onset of decomposition increases escape time in the event of a fire. When the material starts decomposing, it releases gases into the atmosphere, which, when mixed with sufficient amount of oxygen and exposed to heat, will ignite. Delay of onset of degradation therefore translates to a delay in the ignition time. This delay in ignition time, however small, can mean the difference between life and death.

The char performance of the 0.45 vol.% clay sample is similar to that of the 0 vol.% clay sample, but the former also shows a remarkable increase in the onset temperature of degradation. This goes to show that with just a small quantity of clay added to the PI-NGS nanocomposite, the thermal stability of the system can be enhanced, while saving on cost and preserving and/or improving on the properties of the polymer, because the dispersibility of the filler in the matrix is still excellent at low loadings of clay.

**Figure 4:**
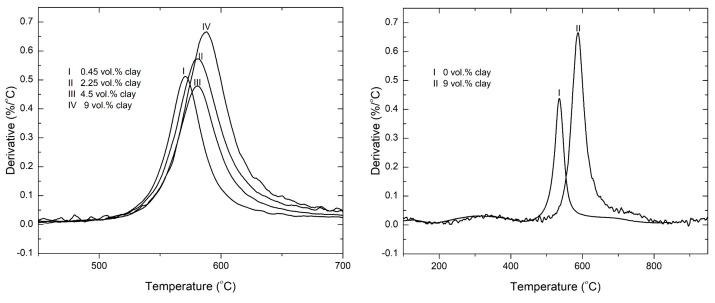
Derivative TGA curves of PI-NGS-Clay nanocomposites in nitrogen atmosphere at a heating rate of 30 °C/min.

The temperature at maximum rate of decomposition (T_max_) is observed to have an overall shift towards higher temperatures. This is shown in Figure 4 below. A shallow peak is observed between 200–400 °C in all the samples. This is the weight loss resulting from the imidization reaction of the polymer matrix [43]; this leads to the release of water upon exposure to higher curing temperatures, resulting in the weight loss observed.

**Figure 5:**
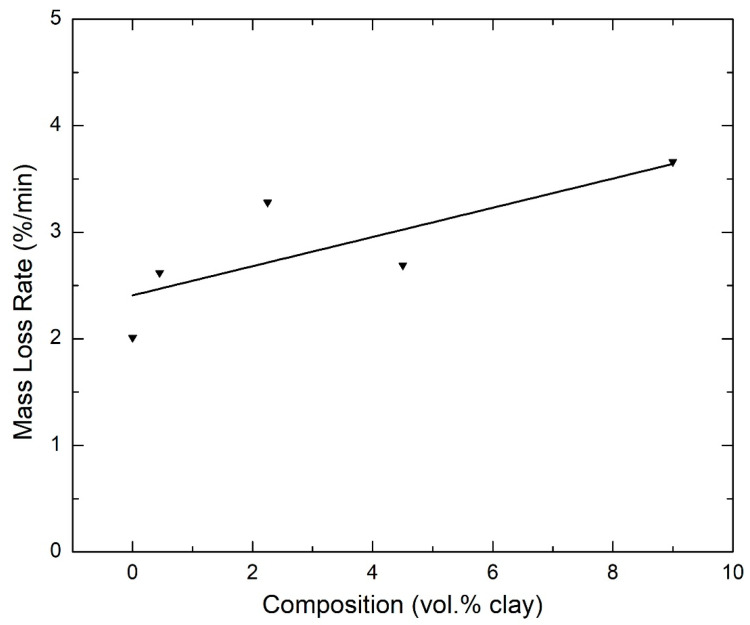
Variation of the rate of degradation of the polymer matrix as a function of the volume percentage of clay in the nanocomposites (in nitrogen; β = 30 °C/min).

The rate of degradation of the major decomposition peak was obtained and plotted in Figure 5. The rate of degradation is generally higher for the nanocomposites containing clay compared with the 0 vol.% clay sample. The organic modifiers in the clay act as catalysts to the degradation of the polyimide matrix, hence the observed increase in rate of degradation. This was also reported by Zhao et al. [44] in their study of polyethylene/clay nanocomposites. They concluded that organoclay has two opposing effects to the thermal stability of the polymer: the barrier effect improves the thermal stability, while the catalysis effect, which is more prevalent at high clay loadings, decreases the thermal stability. Bordes et al. argued that, at the earlier stages of decomposition, the stacked layered silicates could hold accumulated heat that could further accelerate the decomposition of the polymer matrix as it would act as a secondary heat source [45].

#### 3.2.2. Degradation in Air

**Figure 6:**
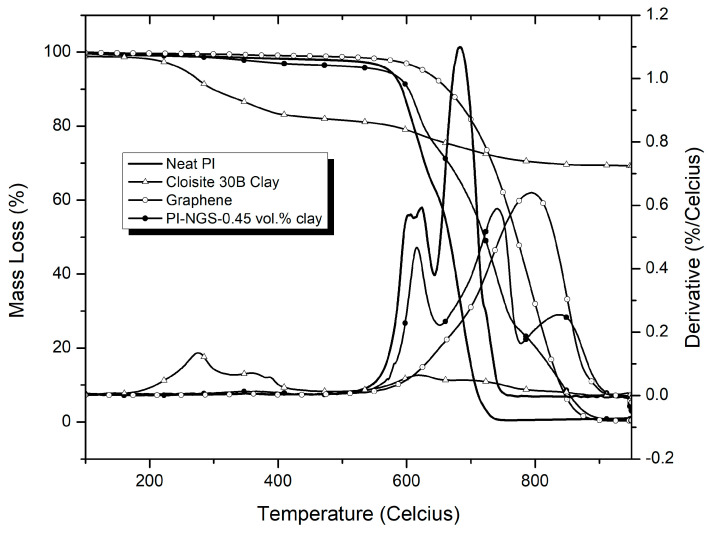
Graph showing the TGA degradation profiles for polyimide, Cloisite 30B organomodified clay, graphene, and PI-NGS-0.45 vol.% clay in air at a heating rate of 30 °C/min.

Figure 6 shows the degradation profiles in air of neat PI, graphene, Cloisite 30B clay, and PI–NGS–Clay nanocomposites containing 0.45 vol.% clay. The organic modifiers in clay start to degrade at lower temperatures (200–400 °C), as was also observed in nitrogen. Clay undergoes minimal degradation in oxidizing atmosphere, with a char retention of approximately 70%. This observation was also reported by Ramani et al. [46].

The PI degrades in multiple steps; degradation of the polymer matrix occurs between 500–700 °C, followed by the degradation of the polymer char, which is complete at ~850 °C. Graphene undergoes one-step decomposition, resulting from the oxidation of carbon. The degradation peak of graphene occurs at approximately 100 °C higher than the degradation peak for the polymer char. A comparison between neat PI and the nanocomposite containing 0.45 vol.% clay shows that the first degradation peak is lower for the nanocomposite, and the second degradation peak is shifted towards higher temperatures.

**Figure 7:**
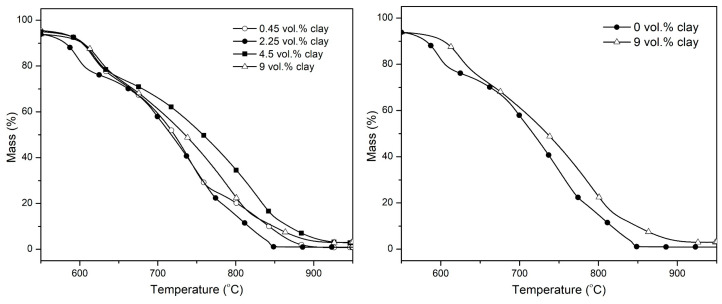
TGA degradation profiles of PI-NGS-Clay nanocomposites in air at a heating rate of 30 °C/min.

TGA mass loss profiles for the PI–NGS–Clay system (Figure 7) shows an overall shift to higher temperatures compared with the sample containing 0 vol.% clay. Clay was shown to undergo minimal degradation in air; it can be deduced that the presence of clay leads to the delayed diffusion of heat and pyrolysis products due to the heat and mass barrier effects observed with layered nanocompounds [47].

**Figure 8:**
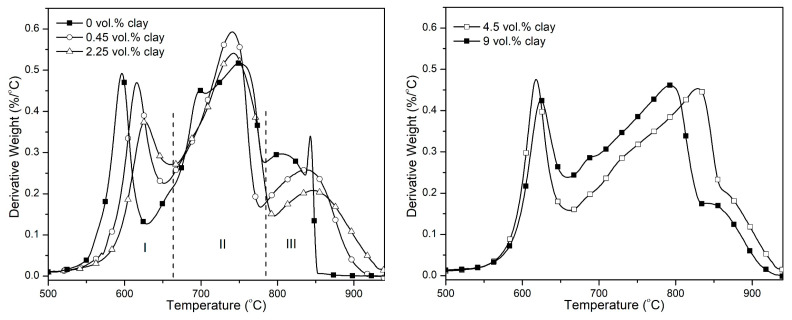
Derivative TGA curves of PI-NGS-Clay nanocomposites showing the degradation regions for (I) imide unit and (II) the polymer char (III) graphene and clay.

The derivative curves shown in Figure 8 show three different regions of degradation. Region I correspond to the degradation of the imide ring, leading to the production of carbon dioxide and other byproducts [48,49]. The polymer char formed in the first stage degrades in region II. This region ranges in temperature from 650–800 °C. In Figure 6, it is shown that the peak degradation temperature of graphene occurs around 800 °C. However, with the addition of clay to the samples, it is observed that the peak degradation temperature of graphene is shifted towards higher temperature by up to 50 °C. This attests to a synergistic interaction between clay and graphene that makes the graphene char more resistant to degradation. With the graphene and clay degrading at higher temperatures, the polymer degradation is slowed down as these fillers shield it from the external heat source.

**Figure 9:**
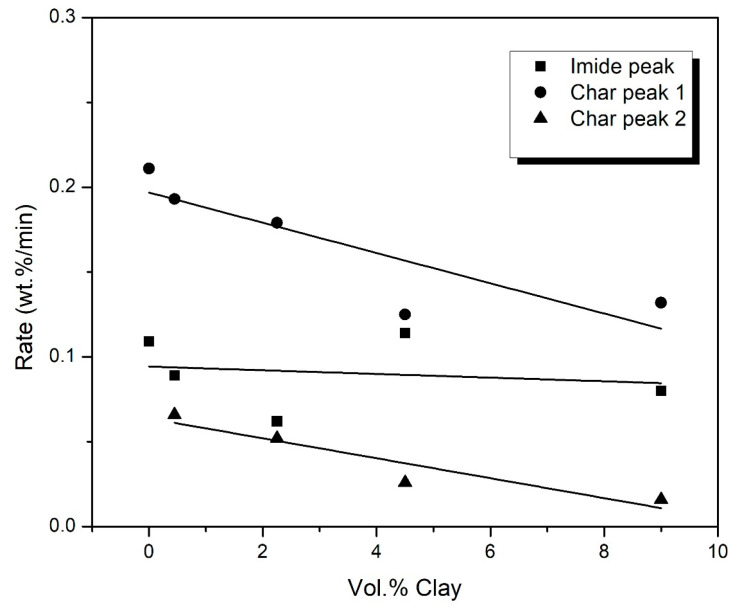
Variation of the rate of degradation of the imide unit and the char for PI-NGS-Clay nanocomposites, as a function of the vol.% clay.

The rate of degradation of the three regions in air is shown in Figure 9. The rate of degradation of the imide peak is observed to decrease at 0.45 vol.% and 2.25 vol.% clay. However, at higher loadings of clay, the rate is observed to slightly increase beyond that of the sample containing only graphene and 0 vol.% clay. As was previously discussed, the organic components of the modified clay may be acting as catalysts to the degradation of the underlying polymer. The rates of degradation of char peaks 1 and 2 (regions I and II in Figure 7) decrease as the clay loading increases. Clay does not degrade much beyond 700 °C; therefore, the char peaks comprise graphene and polymer chars mixed with clay residue. The clay residue, consisting of inorganic particles, mostly SiO_2,_ add bulk to the char and fortifies it, making the overall system degrade slower. The graphene char peak (region III) is shown to decrease in height with increasing clay content, confirming the effect of clay on the degradation of graphene char. The decrease in the rate of degradation of graphene is therefore as a direct result of the increasing clay content.

### 3.3. Differential Scanning Calorimetry

**Figure 10:**
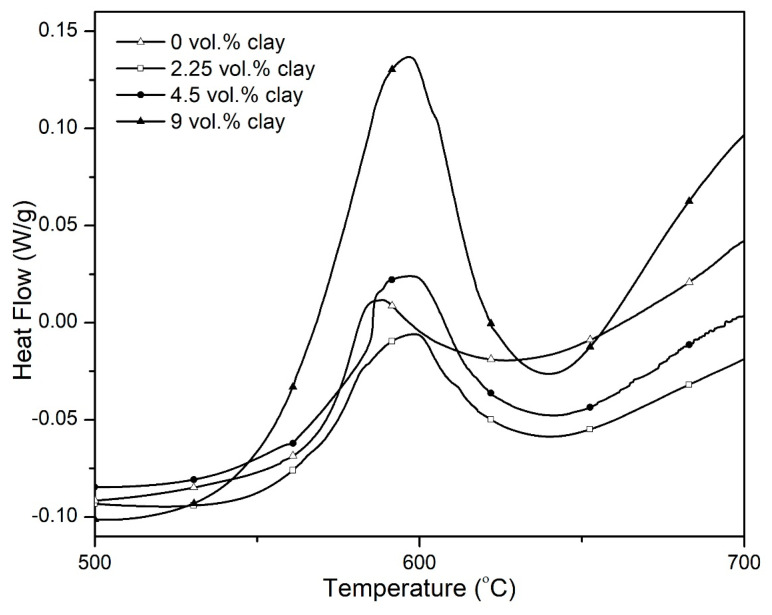
DSC thermograms for the hybrid composites obtained in nitrogen atmosphere at a heating rate of 10 °C/min.

**Figure 11:**
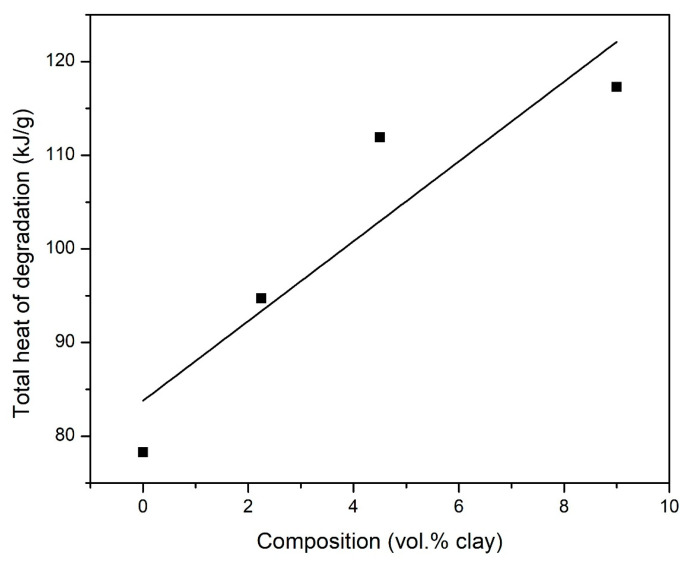
Variation of the total heat released during the degradation of PI–NGS–Clay samples with changing vol.% clay as measured by DSC in nitrogen atmosphere.

DSC thermograms in Figure 10 show the heat flow during the degradation of the composites in nitrogen while Figure 11 shows the total heat released during the degradation process. The total enthalpy change during the degradation of the imide ring was determined by the numeric integration of the DSC peak over the temperature interval. The heat of degradation of the composites is observed to increase with increasing clay loading. This may be due to the high dissociation energy of the SiO_x_ group in clay.

**Figure 12:**
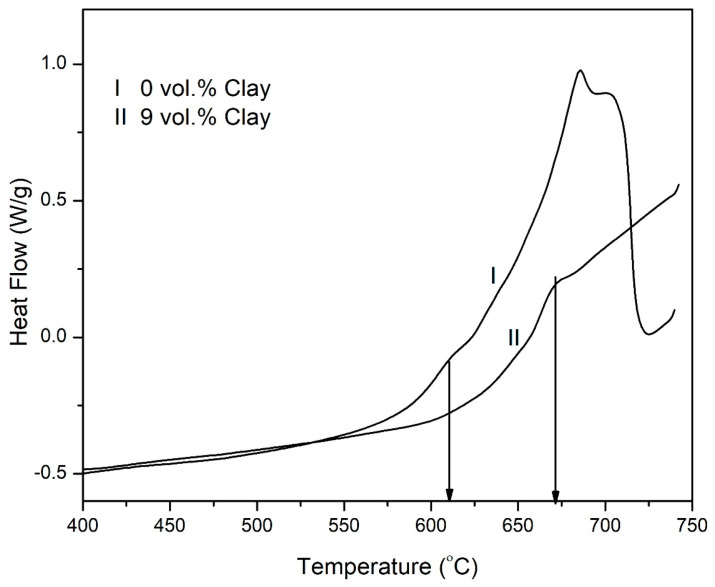
DSC thermograms for the hybrid composites, PI–NGS–Clay, containing, (I) 0 vol.% and (II) 9 vol.% clay in air atmosphere.

**Figure 13:**
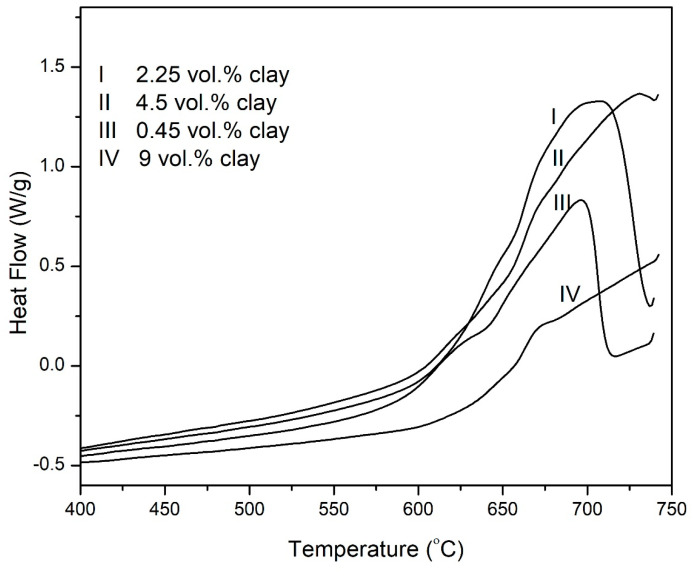
DSC thermograms for the hybrid composites, PI–NGS–Clay, containing, (I) 2.25, (II) 4.5, (III) 0.45, and (IV) 9 vol.% clay in air atmosphere.

The behavior of the samples in air as measured by DSC is different from the nitrogen trends shown above. Figure 12 and Figure 13 show the DSC curves of the samples containing varying compositions of clay. It is evident from the curves that there is a shift in the peak decomposition temperature of the imide peak. There is an increase of approximately 60 °C in the peak decomposition temperature of the imide peak from 0 vol.% to 9 vol.% clay. The char decomposition peaks for the 4.5 vol.% and 9 vol.% samples is beyond the temperature range studied in this work, while that of the 0 vol.% percent falls within the limits (686 °C). This indicates that the chars for the composite containing 4.5 vol.% and 9 vol.% decompose at much higher temperatures. This observation was also confirmed by the TGA results, attesting the improved thermal stability of the PI–NGS–Clay nanocomposite samples.

**Figure 14:**
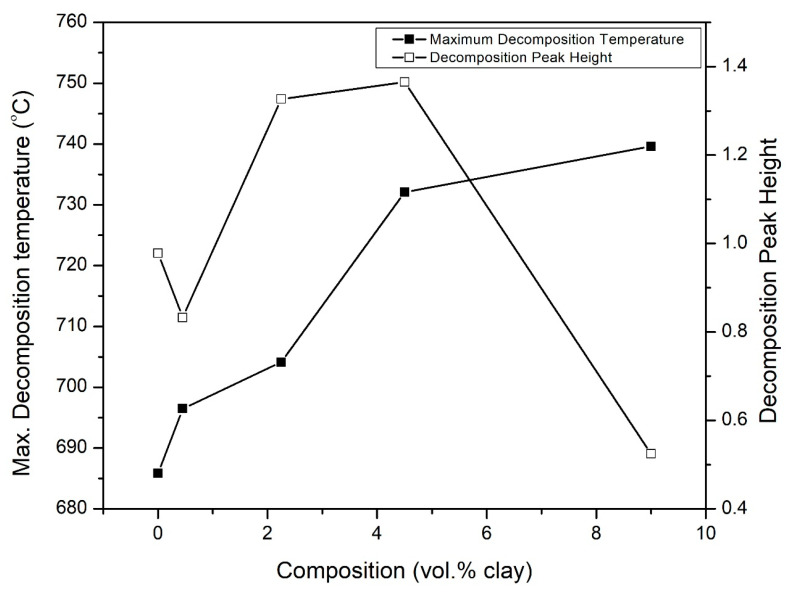
Variation of the decomposition peak height and the maximum decomposition temperature for the hybrid composites as a function of clay vo.%.

In Figure 13, the peak decomposition temperature for the char is shown to gradually increase as the clay vol.% increases in samples. This trend is also shown in Figure 14.

**Figure 15:**
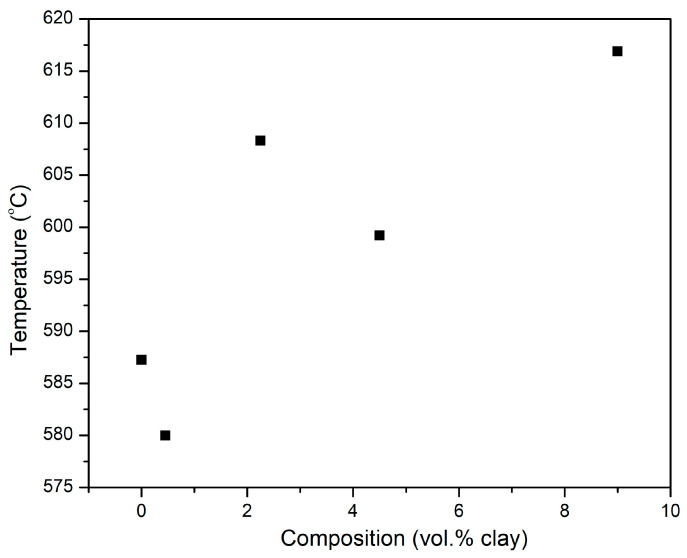
Variation of the onset temperature of decomposition of PI-NGS-Clay nanocomposites as a function of composition.

The temperature at the onset of degradation, shown in Figure 15, is observed to increase and at 9 vol.%, there is about a 5% increase in the onset temperature. This also attests the improved thermal stability. The increase in the onset degradation temperature with increasing clay vol.% follows a similar trend that was shown earlier in Figure 14 for the variation of the maximum temperature for decomposition with vol.% clay. Sachin et al. reported similar findings, in their study of the effect of Cloisite 30B clay on the thermal stability of PMMA-co-BA copolymer [50].

## 4. Conclusions

The thermal degradation and stability of PI–NGS–Clay nanocomposites was investigated. Cloisite 30B clay was observed to maintain high char content (~70%) in both inert and oxidizing atmosphere. The addition of clay to the PI–NGS nanocomposite was shown to increase the onset degradation temperature in both atmospheres, as observed in TGA and DSC. The thermal stability of the system is therefore enhanced by the addition of clay. DSC results show that the char for the hybrid system degrades at much higher temperatures. A synergistic effect is observed between graphene and clay, as the presence of clay tends to shift the temperature of degradation of graphene char to higher temperatures. It was demonstrated that incorporating a small amount of clay into a polyimide–graphene system would enhance the thermal stability of the system. Higher loadings of graphene to achieve greater stability would decrease the ability of the nanofiller to homogenously disperse in the polymer matrix. This would lower certain characteristics of the system. This can therefore be counteracted by using a graphene quantity to the level of optimal dispersibility and then adding small quantities of clay to further enhance the thermal stability of the system without affecting the dispersibility of the nanofillers in the polymer.

## Data Availability

Not applicable.
